# Unsupervised Machine Learning to Identify Separable Clinical Alzheimer’s Disease Sub-Populations

**DOI:** 10.3390/brainsci11080977

**Published:** 2021-07-23

**Authors:** Jayant Prakash, Velda Wang, Robert E. Quinn, Cassie S. Mitchell

**Affiliations:** 1 Laboratory for Pathology Dynamics, Department of Biomedical Engineering, Georgia Institute of Technology and Emory University School of Medicine, Atlanta, GA 30332, USA; prakash1.jayant@gmail.com (J.P.); velda.wang.77@gmail.com (V.W.); rquinn33@gatech.edu (R.E.Q.III); 2 Department of Computer Science, Georgia Institute of Technology, Atlanta, GA 30332, USA; 3 Center for Machine Learning, Georgia Institute of Technology, Atlanta, GA 30332, USA

**Keywords:** Alzheimer’s disease, machine learning, population analysis, risk factors, drug repurposing, clinical trial design

## Abstract

Heterogeneity among Alzheimer’s disease (AD) patients confounds clinical trial patient selection and therapeutic efficacy evaluation. This work defines separable AD clinical sub-populations using unsupervised machine learning. Clustering (t-SNE followed by k-means) of patient features and association rule mining (ARM) was performed on the ADNIMERGE dataset from the Alzheimer’s Disease Neuroimaging Initiative (ADNI). Patient sociodemographics, brain imaging, biomarkers, cognitive tests, and medication usage were included for analysis. Four AD clinical sub-populations were identified using between-cluster mean fold changes [cognitive performance, brain volume]: cluster-1 represented least severe disease [+17.3, +13.3]; cluster-0 [−4.6, +3.8] and cluster-3 [+10.8, −4.9] represented mid-severity sub-populations; cluster-2 represented most severe disease [−18.4, −8.4]. ARM assessed frequently occurring pharmacologic substances within the 4 sub-populations. No drug class was associated with the least severe AD (cluster-1), likely due to lesser antecedent disease. Anti-hyperlipidemia drugs associated with cluster-0 (mid-severity, higher volume). Interestingly, antioxidants vitamin C and E associated with cluster-3 (mid-severity, higher cognition). Anti-depressants like Zoloft associated with most severe disease (cluster-2). Vitamin D is protective for AD, but ARM identified significant underutilization across all AD sub-populations. Identification and feature characterization of four distinct AD sub-population “clusters” using standard clinical features enhances future clinical trial selection criteria and cross-study comparative analysis.

## 1. Introduction

Alzheimer’s disease (AD) is a neurodegenerative condition that is traditionally characterized by tau and amyloid beta protein deposition in neurofibrillary tangles, gradual brain atrophy, and decline in cognitive function [1,2]. In later stages of the disease, people with the condition often experience problems with behavior, language, and disorientation. These symptoms continually worsen over the disease course, progressively worsening until patient death. Despite advancements in medicine leading to decreased deaths from cardiovascular disease and stroke in the United States and other countries, deaths attributed to Alzheimer’s disease continue to increase [2]. 

In response to the continuous increase in mortality related to the disease, the Alzheimer’s Therapeutic Research Institute (ATRI) at the University of Southern California established the Alzheimer’s Disease Neuroimaging Initiative (ADNI) in 2004 with funding from the National Institute of Health (NIH) and various parties in the private sector. Through this initiative, researchers are studying the pathogenesis of Alzheimer’s disease by compiling data on biological markers and neuroimaging taken from patients diagnosed with the condition, as well as from control subjects who exhibit no cognitive deterioration. This data has been made available to investigators at institutions across the globe and has subsequently been interpreted and published in over 1000 studies since the inception of the initiative [3]. Such studies have explored potential early predictors of Alzheimer’s disease, such as hypertension in middle-aged patients [4], cerebrospinal fluid changes correlated with age [5], and gender-specific factors [6]. 

Some researchers have suggested that Alzheimer’s disease can be divided into different subtypes [7]. However, there is no universal sub-population classification system. Alzheimer’s patients have very heterogeneous features, including different onset ages, diverse demographics, assorted clinical phenotypes, multi-factorial pathology, and varied temporal disease progression and response to treatment [8]. The onset age of Alzheimer’s can be divided into early-onset and the more common, late-onset Alzheimer’s disease, the latter of which is defined as occurring in patients over 65 years old [9]. Compared to those with late-onset Alzheimer’s disease, patients with early-onset Alzheimer’s disease displayed greater parietal atrophy, more white matter abnormalities, and less hippocampal volume loss [9]. 

Many studies have discovered various risk factors associated with Alzheimer’s disease including genetic, acquired, and others, although genetics is hypothesized to account for more than 70% of the overall risk [10,11]. Genetic heterogeneity is pervasive in AD, as there is no one single prevailing genotype [10,12]. APP, PSEN1 and PSEN2 genes are well studied in familial AD, whereas late-onset AD is often more associated with alterations in the APOE gene [13]. The acquired risk factors in developing AD are cerebrovascular diseases, diabetes, hypertension, obesity and dyslipidemia [14,15]. Other risk factor studies have tied AD risk to marital status, increased stress, presence of depression, and inadequate sleep [11,16,17,18]. Stress, which is characterized by hyperactivation of the hypothalamic, pituitary and adrenal axis [11], has been shown to increase the accumulation of hyperphosphorylated tau and neurodegeneration in mice [17]. New onset sleep disorders can emerge during the early stages of dementia and worsen its progression [18].

Alzheimer’s disease is diagnosed using a combination of magnetic resonance imaging (MRI) of the brain, clinical cognitive tests that measure functional performance, cerebrospinal fluid (CSF) protein biomarkers, genetic testing, and more. Many such features are included in the ADNI data set, and are measured repeatedly during the patients’ disease course. AD patients have extremely varied baseline features and rates of change in their features with disease progression. The large degree of heterogeneity and complex relationships between biological patterns and clinical manifestations has been a challenge for Alzheimer’s research and treatment. Moreover, the large heterogeneity of patients labelled with the diagnosis of Alzheimer’s amplifies difficulties when comparing results across different cohort studies, as well as identifying clinically meaningful treatment effect sizes in clinical trials. The identification of sub-populations of AD patients based on universally measured clinical features could greatly enhance clinical AD research, clinical trials, and ultimately, personalized patient care.

Unsupervised machine learning techniques provide a promising solution to identifying, quantifying, and characterizing clinical AD sub-populations. Unsupervised machine learning techniques, such as clustering and association rule mining, infer patterns from data without reference to known or labelled outcomes. This starkly contrasts with supervised learning, which is the more common form of machine learning that uses labelled outcomes (such as “control” or “Alzheimer’s disease”) to devise a model that can predict the label using explanatory features (or variables). Unsupervised learning identifies associative patterns in the data that help distinguish sub-groups (or, in this case, patient sub-populations) that have similar features. The identification of underlying Alzheimer’s sub-populations based on shared feature patterns could eventually lead to defined, labelled sub-populations. As a foundation, simply quantifying the number of fundamental sub-populations present and their features is extremely helpful for disease characterization and clinical trial patient selection. Given most research studies and clinical trials calculate statistical results using averages derived from patient distributions, it is of utmost importance to identify the make-up of the patients within the distribution based on their sub-population features. A study or cohort sample distribution can be easily skewed based on the sub-populations comprised within it.

Unsupervised clustering algorithms [19] have the capacity to identify complex mathematical relationships between data points and to autonomously sort the data into sub-groups, or clusters, according to those relationships. If the clustering algorithm produces enough clusters that are uniquely distinguishable from one another, observations can be made to draw novel conclusions about the dataset as a whole. Several studies have leveraged clustering algorithms to facilitate the diagnosis of pathology [20,21,22,23]. For example, clustering techniques have been applied to the diagnosis of breast cancer [24], Parkinson’s disease [25,26] headache [27], mental health and psychiatric disorders [28], heart and diabetes diseases [29], and Huntington’s disease [30], among many others. Unsupervised learning techniques have also previously been used specifically with the ADNI dataset in order to assist in the diagnosis and characterization of Alzheimer’s disease. Several studies have explored applying unsupervised learning techniques to identify imaging patterns present in MRI data provided by the ADNI [31,32]. Others have used unsupervised learning techniques to identify biomarkers that could help classify different stages of disease [33]. Very few studies have gone as far as to define clinical populations of patients using clustering algorithms [34,35]. 

The unsupervised machine learning method for identifying co-occurrences, referred to as association rule mining (ARM), was first widely utilized in business enterprises to identify frequent co-occurring purchase patterns via customer segmentation. The most ubiquitous example is seen in online store purchase recommendation systems where items in a customer’s cart are used to suggest additional purchases: “customers like you also bought…”. However, in the biomedical data science setting, ARM can identify feature co-occurrences (drugs, antecedent diseases, genotypes, etc.) that provide insight into potential risk or protective factors. ARM can be applied to the entire population as well as to sub-populations identified after clustering to determine how the co-occurrence patterns vary between the overall populations and its sub-population clusters. 

The present study applied clustering algorithms with data from the ADNI database including patients’ sociodemographic characteristics, biomarker values, results of clinical cognitive assessments, and basic MRI imaging features, to identify novel sub-populations of Alzheimer’s patients. In particular, the focus was identifying sub-population baseline features at initial diagnosis, which could help pre-classify patients for better stratification and representation in AD clinical trials. Additionally, ARM was utilized to overlay pharmacologic substance usage, including prescription and over-the-counter drugs or vitamin supplements, on the identified AD sub-populations. ARM ascertained whether specific pharmacologic usage classes are associated with specific AD sub-populations, providing insight as to whether such substances may increase AD risk or, conversely, provide neuroprotection.

## 2. Materials and Methods

The methods include a description of the Alzheimer’s Disease Neuroimaging Initiative (ADNI) data set, feature extraction from the ADNI data set, unsupervised clustering to ascertain optimal patient clusters, and association rule mining to find combinatorial pharmaceutics or supplements that are over- or under-represented in the analyzed AD patient sub-populations.

### 2.1. ADNI Dataset Description

The data utilized to identify the patient sub-populations discussed in this article were obtained from the Alzheimer’s Disease Neuroimaging Initiative (ADNI) database (adni.loni.usc.edu). The ADNI was launched in 2003 as a public-private partnership, led by Principal Investigator Michael W. Weiner, MD. The primary goal of ADNI has been to investigate whether serial magnetic resonance imaging (MRI) and positron emission tomography (PET) scans, genetic assays, biomarkers collected from cerebrospinal fluid and blood samples, and clinical and cognitive assessments can be synthesized to measure the progression of Mild Cognitive Impairment (MCI) and early AD. This data was obtained from a large number of cognitively normal, MCI, and AD subjects that were recruited by over fifty different centers in the United States and Canada with follow-up assessments performed every six months. A subset of the ADNI dataset called ADNIMERGE was analyzed through employing various statistical tests and unsupervised machine learning algorithms in order to derive the patient sub-populations explored in this article. ADNIMERGE includes a reduced selection of the more commonly used variables from the ADNI dataset such as patients’ demographic information, scores on clinical exams, and measures from MRI and PET scans.

Abbreviations for the data set are as follows: AD, Alzheimer’s disease; ADNI, Alzheimer’s Disease Neuroimaging Initiative; ADAS, Alzheimer’s Disease Assessment Scale; ADAS11, Cognitive Subscale (11 items) Alzheimer’s Disease Assessment Scale; ADAS13, Cognitive Subscale (13 items) Alzheimer’s Disease Assessment Scale; ADASQ4, task 4 of the Cognitive Subscale (11 items) Alzheimer’s Disease Assessment Scale; AUROC, area under the receiver operating curve; BCa, bias-corrected and accelerated; cAD, converters to probable Alzheimer’s disease; CDR, Clinical Dementia Rating Scale; CDRSB, Sum of Boxes score of the Clinical Dementia Rating Scale; CI, confidence interval; CSF, cerebrospinal fluid; DIGIT, Digit Span Test score; EN, Elastic Net; FAQ, Functional Activities Questionnaire; GTB, MCI, mild cognitive impairment; MMSE, Mini-Mental State Examination; MRI, magnetic resonance imaging; NB, Naive Bayes; NC, non-converters to Alzheimer’s disease; PET, positron emission tomography; RAVLT, Rey Auditory Verbal Learning Test; RAVLT-F, Forgetting score of the Rey Auditory Verbal Learning Test; RAVLT-I, Immediate score of the Rey Auditory Verbal Learning Test; RAVLT-L, Learning score of the Rey Auditory Verbal Learning Test; RAVLT-PF, Percent forgetting score of the Rey Auditory Verbal Learning Test; TRABCOR, time to complete part B of the trail making test.

### 2.2. Feature Extraction from the ADNI Dataset

Certain features were extracted from the ADNIMERGE dataset to prepare the data for preprocessing. Since the focus of the computational analysis in this article is to identify sub-populations of patients based upon clinical outcomes associated with AD, only data from the records of patients diagnosed with AD was considered for preprocessing. Patients’ Digit Span Test (DIGIT) scores were removed from this data, as these scores were not reported in over 20% of the patient records included in the subset of AD patient data. The following variables from the patient’s records included in the subset were extracted for preprocessing:

- Sociodemographic Characteristics: Patients’ sociodemographic characteristics including gender (PTGENDER), age in years (AGE), years of education (PTEDUCAT), race (PTRACCAT), ethnicity (PTETHCAT), and marital status (PTMARRY).
- Clinical Scales: The Clinical Dementia Rating Scale (CDR) [14] is a test used to classify patients’ cognitive statuses over six domains of cognitive and functional performance: Memory, Orientation, Judgment & Problem Solving, Personal Care, Home & Hobbies, and Community Affairs. The CDR Scale Sum of Boxes (CDRSB) score is obtained by summing the evaluator’s rating from each domain. The Functional Activities Questionnaire (FAQ) [15] is an assessment which rates patients’ ability to independently complete activities of daily living based upon feedback collected from caregivers.
- Cognitive Tests: Cognitive tests are listed in Table 1 with their abbreviations. The Mini-Mental State Examination (MMSE) [16] score is based off a 30-point questionnaire that measures patients’ levels of cognitive impairment. Several scores related to the Cognitive Subscale of the Alzheimer’s Disease Assessment Scale (ADAS) [36], which assesses the cognitive status of patients among the domains of functional memory, language, and praxis, were also included. These encompass the ADAS11, ADAS13, and ADASQ4 scores. In addition to the scores from the MMSE and the ADAS, scores from the Rey Auditory Verbal Learning Test (RAVLT) [37], including the RAVLT Immediate (RAVLT-I), RVALT Learning (RAVLT-L), RAVLT Forgetting (RAVLT-F), and RAVLT Percent Forgetting (RAVLT-PF) scores, time to complete part B of the trail making test (TRABSCOR) were also considered.

### 2.3. Unsupervised Clustering

K-means [19] and density-based spatial clustering and application (DBSCAN) [38] are the two widely used clustering algorithms which are employed in this experiment to detect structures within the dataset. Several dimensionality reductions and visualization algorithms, such as principal component analysis (PCA) and *t*-stochastic neighbor embedding (t-SNE) [39], are utilized to visualize the separation of the dataset in two dimensions. Analyses performed using combinations of dimensionality reduction and clustering techniques were as follows:

- K-means was directly applied to cluster the whole dataset with no prior dimensionality reduction.
- DBSCAN was directly applied to cluster the whole dataset with no prior dimensionality reduction.
- PCA was applied to reduce the dimensionality of the dataset and then k-means was applied to cluster the result of the reduction.
- t-SNE was applied to reduce the dimensionality of the dataset and then k-means was applied to cluster the result of the reduction.
- PCA was applied to reduce the dimensionality of the dataset and then DBSCAN was applied to cluster the result of the reduction.
- t-SNE was applied to reduce the dimensionality of the dataset and then DBSCAN was applied to cluster the result of the reduction.

Note that t-SNE followed by k-means clustering was ultimately found to be the superior method for identifying separable clusters, and thus, reported results focus on the findings of this method. 

All analyses were parallelized on a Linux server equipped with four 64-core Intel Xeon CPU E5-2650 v4 @ 2.20GHz and were performed in Python 3.7, using the implementation of the machine learning techniques available in the Scikit-Learn library.

### 2.4. Statistical Analysis of Clusters

Statistical hypothesis tests were completed in order to determine the significance of each cluster and identify each cluster’s most important features. The post-hoc *t*-test assessed significance differences in continuous features between clusters, whereas the Chi-squared test analyzed significance differences in categorical features between clusters. Tukey’s post-hoc correction was used to modify the significance threshold to adjust for multiple comparisons using a family-wise or overall alpha of 0.05.

- Post-hoc *t*-test—The post-hoc *t*-test compared the mean values of each feature in a particular cluster with the mean values of each feature in the overall dataset. This procedure was repeated for every feature in every cluster. The most significant features in each cluster were determined based upon computed *p*-values that met post-hoc criteria for significance.
- Chi-Squared test—The Chi-squared test compared the mean relative frequency of each categorical feature in a particular cluster and mean relative frequency of that same feature in the overall dataset. This procedure was repeated for every feature in every cluster. The most significant features in each cluster were determined based upon computed *p*-values that met post-hoc criteria for significance.

### 2.5. Association Rule Mining of Patient Medication and Supplement Usage

After obtaining the clinical dataset from the Alzheimer’s Disease Neuroimaging Initiative, all medication names were standardized to a common convention for the sake of analysis. For example, one patient’s medication profile may state ‘vit. c’ while another patient’s medication profile may state ‘vitamin c’. These represent the same substance, and thus, were transformed for analysis to ‘vitamin_c’. Additionally, the pharmacological class of each drug was added so that associations between entire pharmacological classes could be examined versus only individual drugs. For example, the anti-hyperlipidemia drugs, simvastatin and Lipitor, belong to the same pharmacological class. Only the medications of the AD cohort were used to perform association rule mining (ARM). In the present study, there were 424 AD patients across the four studies [40,41,42]. The ARM analysis consisted of two parts: frequent itemset mining and association rule mining, as described below.

Frequent itemset mining is the first step towards identifying association rules. Frequent itemset mining is an important tool because like its name suggests, it is able to identify frequently co-occurring items in a transactional dataset. To better describe frequent itemset mining, we can introduce a set *I*, which contains items; a transaction *T*, which contains a set of items that occur together; and a database *D*, which contains a set of all transactions. A well-known application of frequent itemset mining is called the ‘market basket analysis’ where items frequently purchased together by customers are placed near each other in a physical store or suggested as recommendation in an online market to help increase sales. In this clinical study, each item is a potential medication or patient feature; the features of each patient clinic visit make up a transaction; and all possible medication and features in the dataset make up the database. The goal is to determine which items in *I* occur together within the database *D*, with the emphasis on examining which medications or supplements associate with specific patient AD sub-populations. There are many algorithms that can be utilized for frequent itemset mining: Apriori algorithm, FP-Growth algorithm, EclaT algorithm, TreeProjection algorithm, COFI algorithm, TM algorithm, P-Mine algorithm, LP-Growth algorithm, Can-Mining algorithm, and EXTRACT algorithm [43]. Apriori was used for this study since it is a classical algorithm for mining frequent itemsets and relevant association rules.

Apriori operates on transactional databases, and each transaction is considered as a set of items (an itemset). It uses a “bottom up” approach where frequent subsets are extended one item at a time (known as candidate generation). The Apriori algorithm is an iterative process that has four phases: candidate generation, joining, pruning (for itemsets of 3 or more), and evaluation. The Apriori algorithm begins by generating all one-element itemsets (singletons) and determining their support. If the single items do not meet the minimum support threshold, they are discarded. Next, all of the frequent singletons are joined with each other to produce itemsets consisting of two items. Each of these two-itemsets are evaluated to determine if their combined support exceeds minimum support. The infrequent itemsets are once again discarded. This process can continue to produce as many antecedents and consequents as needed [44]. Overall, the Apriori algorithm dictates that all subsets of a frequent itemset must be frequent, and similarly, the supersets of any non-frequent itemset must be infrequent as well. Due to the overall patient sample size of the present study, Apriori frequent itemset generation was limited to two joined singletons.

Frequent itemsets, by themselves, are non-directional. Thus, directionality must be assessed to determine which drugs are associated with specific AD sub-populations. Association rules provide directionality to frequency itemsets. Association rules are if-then rules (X→Y.) and have two parts: an antecedent and a consequent. As the name implies, ARM can only identify associations and not causation. Nonetheless, the identification of associations in a sparse data set can spur the development of hypotheses that can be subsequently experimentally assessed for causation.

Association rule mining generates many rules. Thus, statistical parameters must be set to produce effective and informative association rules. The most well-known measures are support and confidence, which provide quantitative minimum thresholds for assessing the validity and utility of a rule. Support is a measure of how frequently an itemset appears in the dataset. supp(X) = (number of times X occurs in a transaction)/(total transactions) and supp(X→Y) = supp(Y→X) = support(X∪Y). Confidence is used to determine directionality because it indicates the likelihood of item Y occurring when item X occurs. conf(X→Y) = supp(X∪Y)/supp(X). Confidence can be viewed as a conditional probability P(Y|X): the probability of item Y appearing in transactions given the transaction already contains X. However, the limitation of the confidence measure is that it only considers the popularity of itemset X, but not Y. If Y occurs as frequently as X, there will be a greater probability that a transaction containing X will also have Y, resulting in a greater confidence. Therefore, to account for this limitation, lift is used as another measure of effectiveness. lift(X→Y) = supp(X∪Y)/(supp(X) × supp(Y)). Lift indicates the likelihood of itemset Y occurring when item X occurs, while taking into account the frequency of Y. A lift value greater than 1 indicates that Y is likely to occur with X, whereas a value less that 1 signifies that Y is unlikely to occur when X occurs. A fourth measure is conviction: conv(X→Y) = (1 − supp(Y))/(1 − conf(X→Y)). Conviction compares the probability that X occurs without Y if they were dependent on the actual frequency of X without Y. Lastly, leverage measures the difference of X and Y co-occurring in the dataset and what is expected if X and Y were statistically dependent: lev(X→Y) = supp(X→Y) − supp(X) × supp(Y). 

The present study primarily utilized support, confidence, and lift to assess association rules. Using a minimum threshold of 0.001 for support, 0.7 for confidence, and 1.0 for lift, 246 association rules were generated to determine association rules for medication or supplement usage among a specific patient sub-population (cluster 0, 1, 2, or 3).

## 3. Results

The objective of this study was to approximate how many sub-groups or “sub-populations” are present within Alzheimer’s disease and to determine their defining features based on readily-available clinical measurements. Results begin with descriptive analyses of the utilized ADNI dataset. Next, the unsupervised clustering of patient features is presented to predict the optimal number of clusters, which represent separable patient sub-populations, and to quantitatively compare and contrast their clinical features. Subsequently, association rule mining (ARM) is used to identified pharmaceutics or supplement usage that indicate either an increase in potential disease risk or, conversely, infer possible neuroprotection. Finally, pharmaceutics and supplement frequencies and co-occurrences from ARM are overlaid on the identified separable patient sub-populations to assess their overlap with disease severity and progression. 

### 3.1. Descriptive Analysis

Descriptive analysis was preformed upon the dataset prior to preparing it for further analysis during the experiments. The statistics related to the categorical features presented in Table 2 provide a sociodemographic characterization of the overall AD patient population. In addition, the statistics related to the continuous features provided in Table 2 describe the distributions of the studied patients’ brain volumes and cognitive test results. A few global trends were noted among the variables related to brain volume and cognitive tests: the volumes of patients’ hippocampus regions appear to decrease over disease time course and the patients’ MMSE decrease over time. Notably, the distributions of the hippocampus region volumes and MMSE scores approximate a Gaussian distribution.

During the preprocessing of the data, signs on data points expressed as inequalities were excluded for the sake of processing (e.g., a biomarker value expressed as ‘<200’ would be converted to ‘200’). Data points recorded as categorical values were converted into integers to represent each category. Skewness was removed from the data by applying logarithm, cube root, and piecewise linearization functions. The dataset was standardized in order to optimize the running of the model. Some missing data observations were imputed using the standard k-nearest neighbors technique (KNN) to preserve sample size for this exploratory study examining associative patterns.

### 3.2. Identifying Optimal Number of Patient Clusters

**Figure 1:**
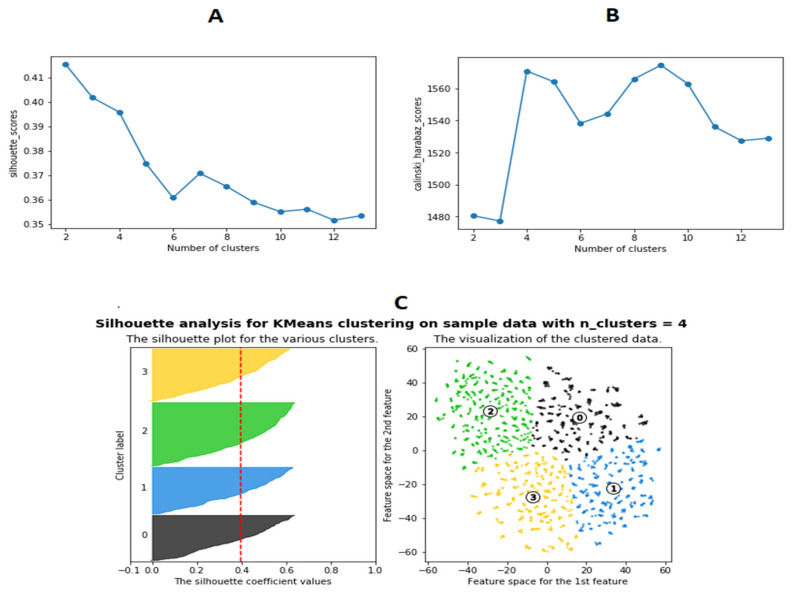
Assessing the optimal number of ADNI Alzheimer’s patient clusters (i.e., AD patient sub-populations) using Silhouette analysis: (**A**) Silhouette scores distributed over cluster sizes; (**B**) Caliniski–Harabaz scores distributed over cluster sizes; (**C**) Silhouette plot and distribution of the data after being clustered with the k-means algorithm. The optimal number of Alzheimer’s patient clusters (or separable sub-populations) was determined to be four.

**Figure 2:**
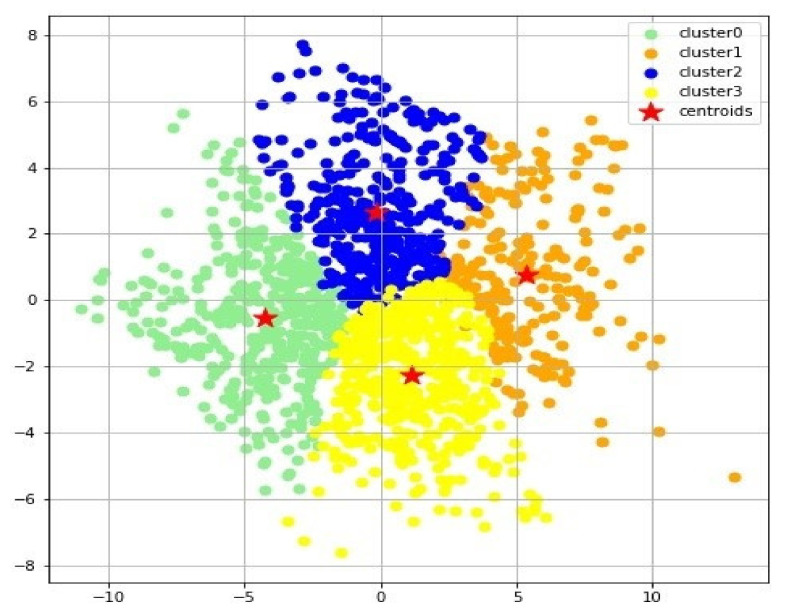
Distribution of ADNI Alzheimer’s patients after being partitioned into four clusters or separable sub-populations (cluster 0, cluster 1, cluster 2, cluster 3) using t-SNE dimensional reduction followed by k-means clustering with k = 4. Red star(s) represents the centroid of each cluster.

A variety of clustering techniques or dimensionality reduction combined with clustering were tried as noted in Methods and Materials. However, the k-means clustering of the t-SNE results were found to be superior based on quantitative ability to optimally produce distinct, separable clusters. Analyses were conducted to determine the optimal number of clusters (the value of “k” for k-means). Silhouette (Figure 1A) and Calinski–Harabasz (Figure 1B) scores for the analysis results were plotted for the number of clusters ranging from 2 to 12. Both of these scores were indicative of the average distances between each respective data point and cluster in the analysis. The number of clusters at which the Silhouette and Calinski–Harabasz scores fluctuate the most typically indicates the maximum number of distinctive, separable clusters. Results indicate that the optimal number of clusters is four (Figure 1C). The k-means clustering algorithm was used to form 4 clusters from the t-SNE reduced dataset (Figure 2).

### 3.3. Comparing Features between Clusters

The 10 most significant continuous features were obtained from the post-hoc *t*-test. The most significant categorical features identified by the Chi-squared test were also recorded. Once these dominant features were identified, the average fold change was calculated. The average fold change was defined as the change in the average value of a feature in a cluster compared to the value of that feature in the overall dataset. From those average fold change values, the percent fold change was calculated for each feature (Table 3).

**Figure 3:**
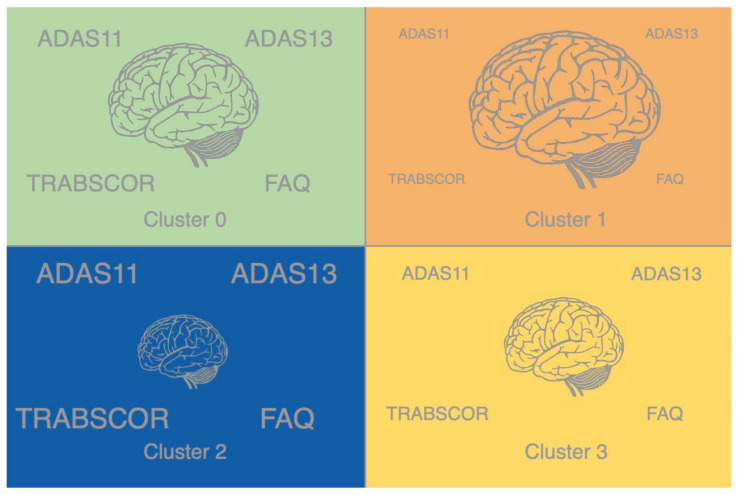
Visualization of the relative magnitudes of the clusters’ average brain volumes and cognitive test scores. Relative sizes indicate higher or lower metric values. Clusters ordered from highest to lowest average brain volume is: cluster 1 > cluster 0 > clusters 3 > cluster 2. Clusters ordered from best to worst cognitive performance: cluster 1 > cluster 3 > cluster 0 > cluster 2.

Cluster 1 features the highest average brain volumes and the best cognitive performance, including the lowest ADAS11, ADAS13, TRABSCOR, and FAQ scores. Cluster 2 features the lowest average brain volumes and worst cognitive performance, including the highest ADAS11, ADAS13, TRABSCOR, and FAQ scores. Cluster 3 features the second-lowest average brain volumes and second-best cognitive scores. Cluster 0 features the second-worst ADAS11, ADAS13, TRABSCOR, and FAQ scores and second-highest brain volumes. These relationships are depicted by the information visualization in Figure 3. Clusters ordered from highest to lowest average brain volume: cluster 1 > cluster 0 > clusters 3 > cluster 2. Clusters ordered from best to worst cognitive performance: cluster 1 > cluster 3 > cluster 0 > cluster 2.

**Figure 4:**
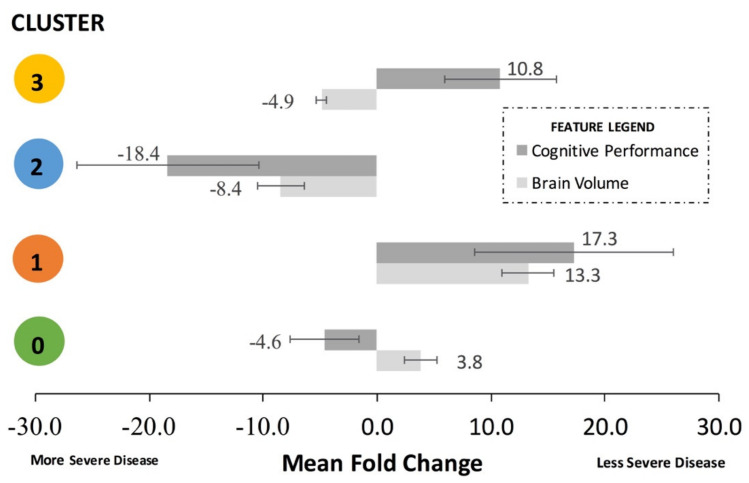
Aggregate standardized trends of brain volume and cognitive performance. Metrics from Table 3 were separated into features measuring brain volume or features measuring cognitive performance. To calculate the represented mean fold change of each cluster, the actual signs of the feature fold changes in Table 3 were subsequently standardized to align with either “less severe disease”, represented by an average positive fold change (higher brain volume or better cognitive performance), or “more severe disease”, represented by a negative fold change (lower brain volume or worse cognitive performance). Error bars represent the standard deviation of the standardized mean fold change of the cluster compared to the whole dataset. Cluster 1 had the least severe disease (highest brain volume and best cognitive performance), whereas cluster 2 had the most severe disease (lowest brain volume and worst cognitive performance). Clusters 0 and 3 represent different mid-severity sub-populations. Cluster 0 had relatively higher brain volume but relatively worse cognitive performance, whereas cluster 3 had better cognitive performance but lesser brain volume.

It is helpful to combine the brain volume feature fold changes and the cognitive performance feature fold changes for each cluster to assess aggregate trends. To do this, the signs of the cognitive performance fold changes must be standardized. For example, a lower mean MMSE score and a higher ADAS11 both correspond to a decline in cognitive performance; thus, their absolute signs from Table 3 must be standardized to represent the same direction of effect on cognitive performance. Thus, to calculate the mean fold change for Figure 4 only, the actual signs of the feature fold changes from Table 3 were subsequently standardized to align with either “less severe disease”, represented by a mean positive fold change, or “more severe disease”, represented by a mean negative fold change in Figure 4. The standardized mean fold change was defined as the change in the standardized mean brain volume features and the standardized mean cognitive performance in a cluster compared to the overall dataset. 

Cluster 1, representing the least severe disease, had a relative 17.3-fold mean greater cognitive performance and 13.3-fold mean greater brain volume compared to the remainder of the dataset. Cluster 0, representing one sub-population of mid-severe disease, had a relative 4.6-fold mean lesser cognitive performance and 3.8-fold mean greater brain volume and compared to the remainder of the dataset. Cluster 3, representing a second sub-population of mid-severe disease, had a relative 10.8-fold mean greater cognitive performance and 4.9-fold mean lesser brain volume compared to the remainder of the dataset. Cluster 2, representing the most severe disease, had a relative 18.4-fold mean decrease in cognitive performance and relative 8.4-fold mean decrease in brain volume compared to the remainder of the dataset.

### 3.4. Association Rule Mining of Pharmaceutic Combinations

The association rule mining (ARM) technique successfully identified association rules between medication usage, AD diagnosis, and AD sub-population. Using a minimum threshold of 0.001 for support, 0.7 for confidence, and 1.0 for lift, 246 association rules were generated. These rules include diagnoses other than AD (CN, EMCI, LMCI, SMC) and both directionalities (prescribed medication diagnosis and diagnosis prescribed medication). There were 8 association rules generated for medication usage and AD, which can be described by the following categories: AD therapies, vitamins, statins, and anti-depressants, which are further discussed below.

#### 3.4.1. Association Rules for Individual Drugs

Association rules were found between drugs commonly prescribed to treat Alzheimer’s disease and an AD diagnosis. This result was expected, and not surprisingly, the rules comprising AD treatment-related drugs had the highest support, confidence, and lift. Aricept, Namenda, and donepezil were associated with AD, from lowest to highest support, respectively. Namenda has a higher confidence, lift, leverage, and conviction that both Aricept and donepezil. Aricept is the brand name drug for donepezil, but both appeared in the results because proprietary names and common names in the dataset were kept when standardizing the drug names in the data engineering/pre-processing step in order to assess any potential differences based on drug brand versus drug class. The rule {Aricept:AD} applied to 244 patients, {Namenda:AD} applied to 226 patients, and {donepezil:AD} applied to 49 patients.

Association rules were also generated for vitamins and the diagnosis of AD, specifically vitamin C and vitamin E. Vitamin C had the second-highest support behind Aricept and Namenda. Vitamin E had lower support but a higher confidence, lift, leverage, and conviction than vitamin C. The rule {vitamin C:AD} applied to 68 patients and {vitamin E:AD} applied to 79 patients. There was no association rule between vitamin D and AD. However, the ARM analysis in the present study did establish an association rule between vitamin D and cognitively normal (CN) patients.

Association rules were found between statins and an AD diagnosis, specifically simvastatin and Lipitor. Simvastatin had a greater support than Lipitor, though Lipitor had a higher confidence, lift, leverage, and conviction. The rule {simvastatin:AD} applied to 62 patients and {Lipitor:AD} applied to 53 patients. Note that both drugs are used to treat hyperlipidemia and belong to the same class.

Finally, a strong association rule was found between the antidepressant, Zoloft, and the diagnosis of AD, and this rule applied to 48 patients.

#### 3.4.2. Association Rules for Pharmacologic Drug Classes

In the data engineering/pre-processing phase, the pharmacological classes of each drug were added as an additional column. The Apriori algorithm was run on the classes to determine mechanisms of action in order to gain a broader understanding of the relationships between drugs and AD. Because the drug classes roughly correspond to the drug indication, it is intuitive that AD symptom therapeutics have the highest support, confidence, and lift. However, other drug classes not necessarily utilized for AD-specific symptomology associated with AD as well. The primary drug classes that were found to be the most associated with AD were (in descending order for support): cholinesterase inhibitors, NMDA receptor antagonists, serotonin reuptake inhibitors, HMG-coA reductase inhibitors, vitamin c, ascorbic acid, vitamin B12, and vitamin E.

#### 3.4.3. Overlaying Pharmacologic Associations with the Cluster Features

**Figure 5:**
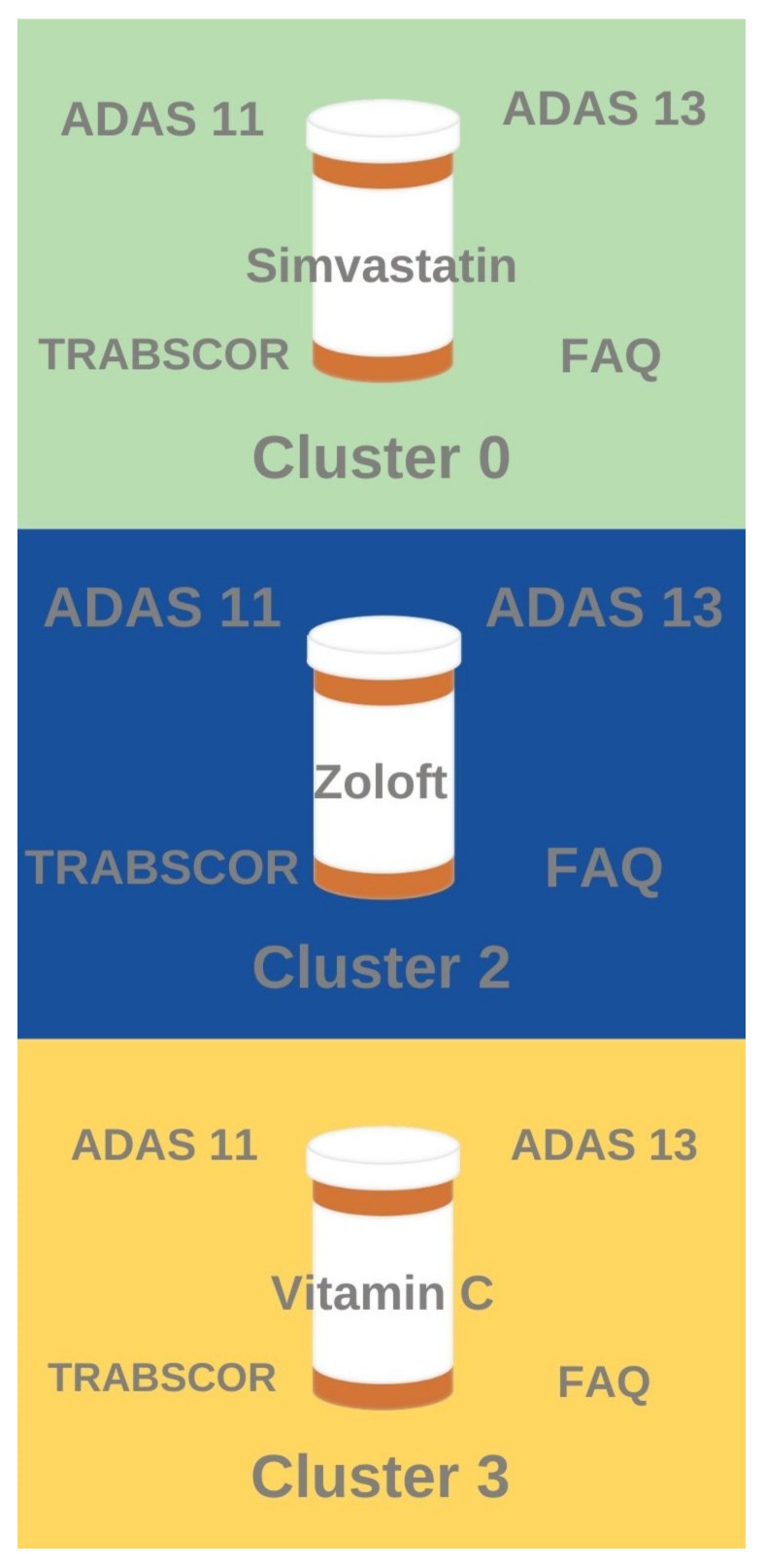
Visualization of the association rules found between AD sub-populations (clusters) and prescribed drugs or supplements. The relative font size of the cognitive test scores indicate relative feature values. Notably, no major association rules were found between pharmacological substances and cluster 1 (least severe disease).

Several notable associations were found between the clustering results and the drug association rule mining results (Figure 5). Patients who have taken anti-depressants like Zoloft exhibited similar high ADAS11, ADAS 13, TRABSCOR, and FAQ assessment scores to patients and tend to belong in Cluster 2 (most severe disease with lowest brain volume and worst cognitive scores). Likewise, patients who have taken anti-hyperlipidemia drugs like Simvastatin tend to belong to Cluster 0 (mid-severity sub-population with relatively higher brain volume but relatively lesser cognitive performance). Patients who have taken Vitamin C tended to fall in Cluster 3 (mid-severity sub-population with relatively higher cognitive performance but relatively lower brain volume). Interestingly, no significant frequent drug classes were identified for Cluster 1, the AD sub-population with the least severe disease (highest brain volume and best cognitive performance).

## 4. Discussion

Unsupervised clustering identified the separation between standard clinical features of patients to produce an optimal number of clusters, which was determined to be four clusters (cluster 0, cluster 1, cluster 2, cluster 3). Cluster 1 represented least severe disease (highest brain volume, best cognitive performance), whereas cluster 2 represented most severe disease (lowest brain volume, worst cognitive performance). Cluster 3 represented a mid-severity sub-population with relatively better cognitive performance and lower brain volume, whereas cluster 0 represented a mid-severity sub-population with relatively lower cognitive performance and relatively higher brain volume. Association rule mining identified that patients treated with anti-hyperlipidemia drugs tended to fall in cluster 0 (mid-severity, higher brain volume sub-population), patients treated with anti-depressants tended to fall in cluster 2 (most severe disease), and patients treated with vitamin c tend to fall in cluster 3 (mid-severity, better cognition sub-population). No major drug associations were found with cluster 1 (least severe disease).

### 4.1. Comparing Alzheiemer’s Disease Clustering Results

A few other approaches have been taken in order to apply clustering algorithms to identify clinical sub-populations of patients with Alzheimer’s disease. These other studies had different objectives, but nonetheless, some comparisons can be drawn to the present work. The key conclusion is that every study has identified separable patient populations; however, not every study used the same features. This conclusion underscores the vast heterogeneity in the Alzheimer’s population and the need to define standardized sub-populations that enable more precise prediction of disease progression, comparison of different AD patient cohorts (by being cognizant of the features of the cohort distribution), and clinical trial patient selection.

One novel experiment separated patients based upon gender and performed multi-layer clustering within each gender group by utilizing the Random Rule algorithm to generate example similarity tables, calculating clustering-related variability scores from those tables, and partitioning the data accordingly [34]. Another study partitioned ADNI data by employing k-Medoid clustering [35]. While these works included pre-Alzheimer’s disease populations in their analyses, used different clustering algorithms, and generated different numbers of clusters compared to the current study, parallels can be drawn between the results of those works and the findings of this study.

The study that separated the patients by gender [34] produced six clusters: two female clusters and four male clusters. For female patients, cluster F0 represented those with no or mild dementia, while cluster F1 represented those with significant cognitive challenges. For male patients, clusters M0A and M0B represented those with no or mild dementia, while clusters M1 and M2 represented those with significant cognitive challenges. The male clusters that represented the same disease stage were split based upon exhibiting different significant features. There are a few notable similarities between these clusters and the clusters found in the current study. Cluster 1 features high baseline FDG, hippocampus, and whole brain volume values similar to cluster F0 and M0A. Cluster 2 features high ventricle, low FDG, low hippocampus, and low ABETA values similar to cluster M2. Cluster 3 features low baseline FDG, hippocampus and whole brain volume values as well as a high tau value. These trends are also noticed in clusters M1 and F1.

Similar to the gender-centric study, the study that employed k-medoid clustering [35] also generated six clusters: “Healthy”, “Affective Mild Cognitive Impairment (MCI)”, “Anosognosia dementia”, “Worried Well”, “Uncompensated MCI”, and “Insightful dementia.” A few similar trends were noticed between these clusters and the clusters found in the present study. Concerning cognitive assessment scores, the “Affective Mild Cognitive Impairment (MCI)” cluster and cluster 2 both featured slightly lower RAVLT_per_forgetting values than average. The “Worried Well” cluster and cluster 1 both had significantly less ADAS11 values than average as well. The “Affective Mild Cognitive Impairment (MCI)” and “Worried Well” clusters and cluster 3 also exhibit slightly elevated MMSE scores compared to the average score. As for brain volume metrics, a few similarities were noted surrounding the Fusiform and WholeBrain values. The “Healthy”, “Affective Mild Cognitive Impairment (MCI)”, and “Worried Well” clusters, as well as clusters 0 and 1, all feature higher-than-average Fusiform values. In contrast, the “Uncompensated MCI” and “Insightful dementia” clusters, in addition to clusters 2 and 3, all feature lower-than-average Fusiform values. The “Affective Mild Cognitive Impairment (MCI)” cluster and cluster 1 both exhibit higher-than average WholeBrain values. 

Cross-referencing these works assists with the clinical characterization of the clusters found by the present study. When attempting to determine whether the clusters represent unique clinical sub-populations, taking the brain reserve hypothesis into account becomes critical. The brain reserve hypothesis suggests that brains with larger volumes better withstand pathological damage before exhibiting cognitive decline [45]. Prior research [45] supports this hypothesis through presenting the relationship between brain atrophy and cognition. This raises interest in further analyzing the relationships between clusters in terms of trends in brain volume measurements and cognitive test scores.

In the present study, patients in clusters 0 and 1 exhibit higher-than-average brain volume metrics. The biomarker trends in these clusters also correlated with those of clusters representing pre-Alzheimer’s disease patients from previously referenced studies. Considering the ADNI already classified all of the patients studied in this current work as being diagnosed with Alzheimer’s disease, this may suggest that cluster 1 represents patients with early stage or mild Alzheimer’s disease. Meanwhile, patients in clusters 2 and 3 feature lower-than-average brain volume metrics. The patterns of biomarker values in these clusters also correlated with functionally worse disease. Taking into account the brain reserve hypothesis and the overlapping biomarker analysis, such data suggest the overall order of Alzheimer’s disease severity in the present work’s Alzheimer’s patient sub-populations as: Cluster 1 < Cluster 0 < Cluster 3 < Cluster 2. However, given the clusters utilize a substantial number of baseline visit feature values, this conclusion does not necessarily imply a strictly time-based difference in disease staging.

### 4.2. Applications of Association Rule Mining to Alzheimer’s Disease

Medication usage, albeit for antecedent or co-morbid disease or specifically for Alzheimer’s symptom treatment, has been hypothesized to play a role in disease etiology. Not surprisingly, the commonly prescribed AD drugs like Aricept, Namenda, and donepezil were all associated with the diagnosis of AD and were most prevalent in patients in the most severe disease cluster (cluster 2). However, ARM was able to identify rules for several non-AD specific drugs and vitamins, which could shed light on their potential role in the epidemiology and pathology of AD.

#### 4.2.1. Vitamin D Appears to Be Beneficial for Prevention of AD

Vitamin C and vitamin E were found to be associated with the diagnosis of Alzheimer’s disease. It is not surprising that vitamins were found to be associated with AD. Even though it may not help to treat Alzheimer’s directly, those with AD are prescribed vitamins as they may eat less or take in less nutrients as the severity progresses. Additionally, vitamins are essential for brain metabolism and repairing cellular damage. However, it is surprising that Vitamin D was not strongly associated with AD, because many medical professionals prescribe vitamin D to prevent onset and progression of AD since it has been shown that people who low blood levels of vitamin D are more than twice as likely as those with normal vitamin D levels to develop Alzheimer’s disease or other types of dementia. The connection of vitamin D is thought to be related to its role in calcium homeostasis, namely increasing calcium availability. Calcium ions are a vital element for neurotransmitter release. Therefore, a lack of calcium ions can impede the communication between neurons. The lack of a specific strong association rule between vitamin D and any AD sub-population in the present study signifies that perhaps not enough AD patients are taking vitamin D. Conversely, the ARM analysis in the present study did establish an association rule between vitamin D and cognitively normal (CN) patients. Though this association rule cannot confirm a causal relationship, it does support the longstanding inference that vitamin D is likely protective for AD.

#### 4.2.2. Men Suffering from CVD Are More Likely to Develop AD Than Women with CVD

Statins are prescribed for people with high cholesterol to lower their total cholesterol and therefore, lower their risk of cardiovascular diseases (CVD) like heart disease and stroke. The statins that were found to be associated with an AD diagnosis were simvastatin and Lipitor (atorvastatin). The individual identifiers for each patient that the rule applied to were found and their demographics were analyzed. The ADNI study cohorts had approximately equal numbers of men and women in AD groups [40], but out of the 62 patients that the rule {simvastatin:AD} applied to, there were 19 female patients and 43 male patients, meaning of the AD patients prescribed simvastatin, 30.65% are female and 69.35% are male. Out of the 53 patients that the rule {Lipitor:AD} applied to, there were 22 females and 31 males, 41.51% and 58.49%, respectively. Although the percentage of female patients with AD that were prescribed Lipitor is higher than that of simvastatin, the percentage of male patients that the rule applied to was greater for both prescription drugs. There are many literature and clinical studies establishing that CVD, specifically high cholesterol, is a risk factor for AD [46]. The association rules between hyperlipidemia drugs (namely Lipitor and simvastatin) and the diagnosis of AD indicates that either hyperlipidemia itself, or anti-hyperlipidemia drug usage, may contribute to the onset of AD, most likely through a transitive relationship of statins → cardiovascular diseases → Alzheimer’s disease. A causal relationship cannot be established without knowing when patients started on statins in relation to the onset of their AD. However, given that patients taking statins were most prevalent in cluster 1 in the present study, it can be hypothesized that the anti-hyperlipidemia treatments are preventing further cardiovascular comorbidities that would otherwise hasten or worsen diminishing cognition. Further analysis indicates that a greater percentage of AD patients that are prescribed statins are men. Vascular dementia is another type of dementia, and it results from ischemic or hemorrhagic injuries to regions of the brain critical for cognitive functions. Literature supports that side effects of cardiovascular diseases such as atrial fibrillation, heart failure, high blood pressure, atherosclerosis, obesity, and diabetes, are more common among men [47]. Therefore, because CVD is known to be a risk factor for AD and it is known that CVD is more common in men, the data shows that men who have CVD are more likely to develop AD than women. 

#### 4.2.3. Antioxidant Vitamins Do Not Prevent Onset or Progression of AD

The percentage of the general population taking antioxidant vitamins (vitamin C and vitamin E) were compared to the percentage of AD patients taking antioxidant vitamins. In the ADNI studies, there were a total of 424 AD patients [11,13,14], so the percentages of AD patients taking vitamin C and vitamin E were found by dividing the number of patients assigned to each rule by the 424 total AD patients. The rule {vitamin C:AD} applied to 16% of the AD cohort and the rule {vitamin E:AD} applied to 18.6% of the AD cohort. Association rules were found between vitamin C and vitamin E and a diagnosis of cognitively normal (CN) and low mild cognitive impairment (LMCI), the latter which often progresses toward mild and moderate Alzheimer’s over time. Despite patients diagnosed as CN and MCI taking vitamins for prophylactic health, there is still a significant association between vitamin C and E and AD. The Vitamin C and E association rules with AD are more prevalent among patients in cluster 3, which is the mid-severity sub-population with higher cognition. This finding signifies that vitamins may play a neutral role in contributing to AD, may not be as helpful as previously thought in ameliorating AD-related neurodegeneration, or that AD patients are simply beginning vitamin supplementation too late to have the desired anti-oxidative stress protective effects necessary to slow or prevent pathological neurodegeneration. Again, because medication start dates are not provided in the present data set, a definitive causal determination cannot be construed from the association identified in the present study’s ARM results. Notably, previous clinical trials and epidemiological data examining the potential impact of antioxidant vitamins on cognitive decline and dementia have also reported mixed findings in the role and/or efficacy of antioxidant vitamins in preventing or slowing dementia [46].

#### 4.2.4. Antidepressants Associated with More Severe AD

A strong association rule was found between antidepressants and AD, namely between Zoloft and the most severe AD sub-population (cluster 2). Interpretation of this association is difficult without knowing when patients started Zoloft treatment (before or after AD onset) and whether the treatment was for antecedent depression that existed prior to the onset of cognitive symptoms of AD or whether Zoloft was prescribed to treat new onset depression that coincided with their AD disease progression. Given the widespread usage of anti-depressants, further research follow-up is necessary to insure that anti-depressants are not further increasing risk of AD in patients using them for depression in the absence of an AD diagnosis.

### 4.3. Comparing Present Study to the State-of-the-Art Machine Learning in Alzheimer’s

Currently, patients are predominantly put into sub-groups based on age of onset and genotype (if available). Key comparable Alzheimer’s disease clustering studies to the present work were discussed in 4.1 Comparing Alzheimer’s Disease Clustering Results. Other types of high-end, state-of-the-art supervised and unsupervised machine learning methods have been performed to assess possible AD sub-populations (e.g., [48,49,50]). While such studies and numerous others that examine multi-modal features are very informative, they mostly rely on novel features that are not available to general Alzheimer’s populations seen outside of research centers or they utilize data mining techniques and software that would not be accessible to the general clinical AD researcher wishing to classify their patients into sub-populations. The relative simplicity of the unsupervised clustering technique presented here and its usage on predominantly universally-available features makes it an ideal method for establishing fundamental sub-populations that generalize well across multiple, large AD populations.

### 4.4. Limitations

The main limitation of the findings in the present work is that unsupervised learning patterns, whether from clustering or association rule mining (ARM), identify association but not causation. Moreover, there is no “ground truth” for the identified patterns since, unlike supervised learning, they are not based on pre-determined labels. Unsupervised learning is also subject to cohort bias or specific sources of bias or noise in the dataset that can impact the generalizability of results. For example, the quantitative fold changes of the clusters could vary with a different patient cohort, although the general patterns with brain volume and cognitive performance are expected to remain consistent given their statistical significance. Additionally, there is no fixed gold standard to measure accuracy of the association rules between prescribed drugs and the diagnosis of Alzheimer’s disease, but this limitation was offset by comparing the ratio of patients to which the rule applied to published general population data. 

### 4.5. Future Directions

The present unsupervised machine learning work identified four optimal clusters using universally-available clinical measurements in the large ADNIMERGE patient cohort. Other high-end machine learning analyses, such as those that utilize specialized electroencephalogram (EEG) [48], biomarkers [49], and multi-modal MRI series [50], are quite valuable. However, for now, they only have utility for patients seen at Alzheimer’s research centers and/or specialists capable of doing complex calculations on large computer servers. The universally available functional measurements and basic MRI series metrics used in the present study are more inclusive to the general Alzheimer’s clinical populations that may not have access to a research clinic. Having sub-population classification algorithms that generalize to as many patients as possible is critical for the usage of future sub-population definitions in research and clinical trials to be effective. Future work should attempt to re-construct the four clusters using the same standardized techniques and same features in cohorts beyond ADNI to confirm the clusters generalize across multiple large Alzheimer’s data sets. While it is expected that the fold changes in features between clusters will quantitatively vary to a degree between cohorts, assessment of fold changes between clusters across multiple cohorts could define quantitative ranges and criteria for formally classifying patients into sub-population clusters at baseline diagnosis. The greatest difficulty will be disambiguating cognitive performance declines due to normative aging versus declines due to Alzhiemer’s pathology. The long-term goal is to develop universal sub-population definitions that are broad enough to generalize across cohorts but, simultaneously, sensitive enough to reduce heterogeneity that clouds research statistical analysis, clinical trials, and personalized patient care. Finally, further research to establish possible causations behind associated common drug and vitamin usage in AD and AD sub-populations is recommended.

## 5. Conclusions

The utilization of k-means clustering in the present study successfully identified four basic clusters that represent separable clinical sub-populations of AD patients using universally available clinical metrics. The four AD sub-populations (i.e. clusters) significantly differ in their whole and specific brain region volumes and cognitive test scores: cluster 1 represented least severe disease (+17.3-fold cognitive performance, +13.3-fold brain volume); cluster 0 represented a mid-severity disease sub-population (−4.6-fold cognitive performance, +3.8-fold brain volume); cluster 3 represented another mid-severity disease sub-population (+10.8-fold cognitive performance, −4.9-fold brain volume); cluster 2 represented the most severe disease (−18.4-fold cognitive performance, −8.4-fold brain volume). Considering the brain reserve hypothesis and overlapping biomarkers, the overall order of sub-population disease severity is likely: cluster 1 < cluster 0 < cluster 3 < cluster 2. Mapping association rules to the AD patient sub-populations indicated that anti-depressants are linked to high ADAS11, ADAS 13, TRABSCOR, and FAQ assessment scores and the worst overall AD outcomes (cluster 2); statins seem to be associated with higher-than-average brain-volume metrics among mid-severity AD patients (cluster 0), possibly protecting against confounding vascular-related dementia; and vitamin C seems to be correlated with better than average cognitive assessment scores among mid-severity AD patients (cluster 3). Notably, no drugs or supplements were strongly associated with AD patients with the least severe disease (cluster 1). ARM results did indicate that vitamin D usage is less among AD patients compared to cognitively normal (CN) patients, indicating that perhaps not enough AD patients are taking vitamin D, as vitamin D is known to reduce the risk of AD. In summary, the four identified clusters provide data-enabled, quantitative evidence for the construction of protocols to classify AD patient sub-populations using traditional, readily available clinical features. Consistent classification of AD patient sub-populations could reduce patient heterogeneity that otherwise confounds the assessment of AD therapeutic efficacy in clinical trials. 

## Figures and Tables

**Table 1:** Abbreviations of cognitive tests for Alzheimer’s disease.

Test Abbreviation	Test Name
ADAS11	Cognitive Subscale (11 items) Alzheimer’s Disease Assessment Scale
ADAS13	Cognitive Subscale (13 items) Alzheimer’s Disease Assessment Scale
ADASQ4	Task 4 of the Cognitive Subscale (11 items) Alzheimer’s Disease Assessment
CDRSB	Sum of Boxes score of the Clinical Dementia Rating Scale
DIGIT	Digit Span Test score
FAQ	Functional Activities Questionnaire
LDT	Logic Memory subtest of the of the Wechsler Memory Scale-Revised
MMSE	Mini-mental state examination
RAVLT	Rey Auditory Verbal Learning Test
RAVLT-F	Forgetting score of the Rey Auditory Verbal Learning Test
RAVLT-I	Immediate score of the Rey Auditory Verbal Learning Test
RAVLT-L	Learning score of the Rey Auditory Verbal Learning Test
RAVLT-PF	Percent forgetting score of the Rey Auditory Verbal Learning Test
TRABSCOR	Time to complete part B of the trail making test

**Table 2:** Descriptive statistics for categorical and continuous ADNI patient features.

	**Categorical Features**		
**Feature**	**Subfeature**	**Proportion**	
PTRACCAT	white	0.933	
	black	0.035	
	asian	0.014	
	more than one race	0.018	
PTGENDER	male	0.55	
	female	0.45	
PTMARRY	divorced	0.037	
	married	0.835	
	never married	0.102	
	widowed	0.038	
PTETHCAT	hispanic/latino	0.009	
	not hispanic/not latino	0.968	
	unknown	0.022	
	**Continuous Features**		
**Feature**	**Mean**	**Variance**	**Standard Deviation**
WholeBrain	966,591	1.32 × 10^10^	115,028
Fusiform_bl	15,523.73	6,954,489	2637
mPACCdigit_bl	−15	11	3.33
MidTemp	16,843	10,707,867	3272

**Table 3:** Top features for each of the four clusters based on analysis with post-hoc *t*-test. (+) denotes an increase, and (−) denotes decrease in percentage compared to the mean values of the whole dataset. Note that ‘_bl’ corresponds to baseline.

Feature	Cluster 0	Cluster 1	Cluster 2	Cluster 3
RAVLT_perc_forgetting	3.55(+)	2.418(−)	4.998(+)	6.958(−)
MidTemp	4.919(+)	14.597(+)	10.152(−)	4.949(−)
MidTemp_bl	3.226(+)	15.898(+)	9.422(−)	5.539(−)
mPACCdigit	10.033(−)	22.818(+)	23.914(−)	17.259(+)
mPACCdigit_bl	11.214(−)	15.934(+)	15.752(+)	14.522(+)
mPACCtrailsB_bl	4.598(−)	20.580(+)	19.909(−)	9.726(+)
Fusiform	5.795(+)	13.635(+)	9.899(−)	5.151(−)
Fusiform_bl	4.922(+)	14.943(+)	10.329(−)	5.045(−)
WholeBrain	2.642(+)	9.930(+)	5.663(−)	4.268(−)
WholeBrain_bl	3.118(+)	10.332(+)	5.850(−)	4.810(−)
ADAS11	3.411(+)	25.079(−)	29.142(+)	15.885(−)
ADAS11_bl	4.338(+)	22.181(−)	28.595(+)	18.606(−)
ADAS13	3.093(+)	20.392(−)	22.367(+)	11.580(−)
ADAS13_bl	2.360(+)	18.605(−)	23.769(+)	14.245(−)
TRABSCOR	3.904(+)	29.043(−)	21.412(+)	3.398(−)
TRABSCOR_bl	3.8176(+)	29.211(−)	24.851(+)	7.344(−)
Hippocampus_bl	1.985(+)	13.518(+)	7.725(−)	4.405(−)
RAVLT_perc_forgetting_bl	5.039(+)	3.684(−)	8.197(+)	10.984(−)
ADASQ4_bl	1.176(+)	9.638(−)	10.816(+)	5.524(−)
MMSE	1.924(−)	11.824(+)	13.608(−)	7.602(+)
MMSE_bl	2.187(−)	5.814(+)	5.960(−)	3.918(+)
RAVLT_immediate_bl	8.610(−)	21.569(+)	22.020(−)	14.853(+)

## Data Availability

Aggregate data is presented in full in the manuscript. Raw data is available via request to the ADNI, http://adni.loni.usc.edu (accessed on 23 July 2021).
